# Structure, Antioxidant Activity and Antimicrobial Study of Light Lanthanide Complexes with p-Coumaric Acid

**DOI:** 10.3390/ma17061324

**Published:** 2024-03-13

**Authors:** Grzegorz Świderski, Ewelina Gołębiewska, Natalia Kowalczyk, Monika Kalinowska, Renata Świsłocka, Elżbieta Wołejko, Urszula Wydro, Piotr Malinowski, Anna Pietryczuk, Adam Cudowski, Waldemar Priebe, Włodzimierz Lewandowski

**Affiliations:** 1 Department of Chemistry Biology and Biotechnology, Bialystok University of Technology, Wiejska 45E, 15-351 Bialystok, Poland; e.golebiewska@pb.edu.pl (E.G.); r.swislocka@pb.edu.pl (R.Ś.); e.wolejko@pb.edu.pl (E.W.);; 2 Department of Water Ecology, Faculty of Biology, University of Bialystok, Ciołkowskiego 1J, 15-245 Bialystok, Polandcudad@uwb.edu.pl (A.C.); 3 Department of Experimental Therapeutics, The University of Texas MD Anderson Cancer Center, 1515 Holcombe Blvd, Houston, TX 77030, USA

**Keywords:** p-coumaric acid, light lanthanides, antioxidant assays, antimicrobial study, MTT test

## Abstract

This paper presents the results of a study of the effects of the lanthanide ions Ce^3+^, Pr^3+^, Nd^3+^ and Sm^3+^ on the electronic structure and antioxidant and biological (antimicrobial and cytotoxic) properties of p-coumaric acid (p-CAH_2_). Structural studies were conducted via spectroscopic methods (FTIR, ATR, UV). Thermal degradation studies of the complexes were performed. The results are presented in the form of TG, DTG and DSC curves. Antioxidant properties were determined via activity tests against DPPH, ABTS and OH radicals. The reducing ability was tested via CUPRAC assays. Minimum inhibitory concentrations (MICs) of the ligand and lanthanide complexes were determined on *E. coli*, *B. subtilis* and *C. albicans* microorganisms. The antimicrobial activity was also determined using the MTT assay. The results were presented as the relative cell viability of *C. albicans*, *P. aeruginosa*, *E. coli* and *S. aureus* compared to controls and expressed as percentages. In the obtained complexes in the solid phase, lanthanide ions coordinate three ligands in a bidentate chelating coordination mode through the carboxyl group of the acid. Spectroscopic analysis showed that lanthanide ions increase the aromaticity of the pi electron system of the ligand. Thermal analysis showed that the complexes are hydrated and have a higher thermal stability than the ligand. The products of thermal decomposition of the complexes are lanthanide oxides. In the aqueous phase, the metal combines with the ligand in a 1:1 molar ratio. Antioxidant activity tests showed that the complexes have a similar ability to remove free radicals. ABTS and DPPH tests showed that the complexes have twice the ability to neutralise radicals than the ligand, and a much higher ability to remove the hydroxyl radical. The abilities of the complexes and the free ligand to reduce Cu2+ ions in the CUPRAC test are at a similar level. Lanthanide complexes of p-coumaric acid are characterised by a higher antimicrobial capacity than the free ligand against *Escherichia coli* bacteria, *Bacillus subtilis* and *Candida albicans* fungi.

## 1. Introduction

Lanthanide complexes with ligands of natural origin are characterised by increased antimicrobial and antioxidant activities compared to the ligands or inorganic salts of these metals. In a study by Zheng et al., 3,5-dimethoxybenzoic acid complexes with terbium, dysprosium, erbium and ytterbium were shown to have higher antimicrobial potential against *Escherichia coli*, *Staphylococcus aureus* and *Candida albicans* than the ligand, which had a low effect on bacteria, as did lanthanide chlorides [1]. Zhao et al. (2017) analysed the effect of complexation with Eu(III), Gd(III), Tb(III), Ho(III) and Er(III) ions on the antimicrobial activity of 2-bromo-5-methoxybenzoic acid [2]. The ligand showed no activity against *Candida albicans*, *Escherichia coli* and *Staphylococcus aureus* strains, while the complexes showed enhanced antimicrobial activity, particularly strong against *Candida albicans*.

In antimicrobial studies of europium, dysprosium and gadolinium complexes with caffeic and p-coumaric acid (p-CAH_2_), complexes showed a higher activity against *E. coli*, *B. subtilis* and *C. albicans* than the ligand or metal chloride [3].

Complexes of erbium, terbium, dysprosium and holmium with 3-bromo-5-iodobenzoic acid show moderate antimicrobial activity against *Candida albicans* and *Staphylococcus aureus* and high activity against *Escherichia coli* [4]. Samarium and terbium complexes with aminobenzoic acid and 2-amino-5-chlorobenzoic acid show higher antimicrobial activity against *S. aureus* (G+) and *E. coli* (G) w bacteria and the fungus *C. albicans* with reference to free ligands [5].

Lanthanide complexes with heteroaromatic ligands (pyridine, pyrazine derivatives) [6,7] and complexes with flavone compounds [8,9,10] or with Schiff bases [11,12,13,14] also show antimicrobial activity.

The increase in antimicrobial activity of the complexed ligand is caused by a decrease in the polarity of the lanthanide ions during complexation due to the transfer of ligand electrons to the free metal orbitals and an increase in the delocalisation of the pi electron system of the ligand. This results in an increase in lipophilicity and the ability to penetrate the lipid membranes of bacterial cells [2,15].

The formation of lanthanide complexes with organic ligands also alters the antioxidant activity. Complexes of La(III), Sm(III), Eu(III), Gd(III), Tb(III) and Dy(III) with salicylaldehyde-2-picolinoylhydrazone (Schiff base) show a higher antioxidant activity in the DPPH assay than the ligand [16]. The same was observed for N,N’-bis(1-naphthalenediamine)-o-phenylenediamine complexes with the lanthanides Dy(III), Tb(III), La(III), Pr(III), Er(III), Sm(III), Nd(III) and Gd(III). The antiproliferative activity tested by the DPPH assay was higher in the complexes than in the ligand [17].

The reason for the higher antioxidant activity of the tested lanthanide(III) complexes compared to the free ligand is the increase in the polarity of the -OH groups. The H atom then exhibits a greater ionisation capacity in the complex than in the free ligand, accelerating the HAT reaction [17].

The antioxidant activity of ligands can also decrease when complexes are formed with lanthanides (also other metals). Complexation of cerium, lanthanum and neodymium with aminoorotic acid results in a decrease in the antioxidant activity of the ligand [18]. Also, in the case of complexes of europium, dysprosium and gadolinium with caffeic acid, a reduced activity of the complexes was observed compared to the ligand [3]. In this case, this was due to the formation of the complex by metal binding to the hydroxyl groups of the aromatic ring of the ligand, which were responsible for the anti-radical properties of caffeic acid. Also, complexation of quercetin with metal ions may reduce its antioxidant activity. The terbium (III) complex with quercetin shows a lower antiradical activity than the free quercetin ligand. The complex is formed by metal attachment to the ligand via hydroxyl groups, causing a decrease in antioxidant activity [19].

Studies have shown that compounds from the phenolic acid group can exhibit higher antioxidant activity when complexed with metals. An example of such a compound is p-CAH_2_, which exhibits a high antioxidant potential [20,21]. Our previous studies have shown that complexes of this acid with transition metals, as well as with some lanthanides, have higher antimicrobial and antioxidant activities than the ligand [3,22]. This paper presents the results of a study of the structure and biological properties of Ce(III), Pr(III), Nd(III) and Sm(III) complexes with p-CAH_2_.

## 2. Materials and Methods

### 2.1. Synthesis

Different amounts of p-CAH_2_ acid (0.001 mol) were dissolved in 10 cm^3^ of a 0.1 mol/dm^3^ aqueous sodium hydroxide solution. The mixtures were incubated in a shaker at 50 °C for 1 h. To the clear solution was added an aqueous solution of lanthanide chloride (cerium, neodymium, praseodymium, samarium) at a concentration of 0.1 mol/dm^3^ in a stoichiometric amount of 3:1 (ligand/metal), and the whole mixture was stirred in a shaker for 2 h at 50 °C. The turbid solutions were left for 48 h to precipitate. The resulting complexes were drained using filters and then washed to elute residual chlorides. The resulting precipitates were dried at room temperature in a desiccator for 72 h and then stored in airtight vessels. The synthesis was carried out three times. The obtained sediments were studied by measuring ATR spectra. The repeatability of the synthesis of the obtained substances was determined, both for the freshly precipitated sediments and for the sediment collected after 48 h. It was not possible to obtain the substance in a crystalline form useful for examining its structure using the X-ray diffraction method.

### 2.2. Elemental and Thermal Analysis

Elemental analysis was carried out on a Perkin Elmer Series Analyser II CHNS (Waltcham, MA, USA). The percentage of carbon and hydrogen in the samples was determined. Measurements were taken three times for each sample and the average results were calculated. Thermal analysis was performed on an STA 600 Frontier thermal analyser (Perkin Elmer, Waltham, MA, USA). Aliquots of samples (approximately 5 mg) were annealed in a ceramic crucible over a temperature range of 40–900 °C in an air atmosphere at a heating rate of 10 °C/min. Thermal decomposition products were analysed based on recorded TG, DTG and DSC curves.

### 2.3. Spectroscopic Study

FTIR spectra were recorded for samples prepared as pressed KBr pellets (1 mg sample per 200 mg KBr) and via the ATR multi-reflectance technique. Spectra were recorded on a Bruker alpha instrument (Billerica, MA, USA) in the range 4000–400 cm^−1^ (number of scans 32, resolution 1 cm^−1^). Raman spectra were recorded for solid-phase samples on a MultiRam instrument from Bruker (Billerica, MA, USA) (measuring resolution 1 cm^−1^, number of scans 124, laser power of 200 mW). Ultraviolet (UV) spectra were recorded on an HACH 2000 DR instrument in the range of 200–400 nm, with a resolution of 0.1 nm. UV spectra were recorded in aqueous solutions at a concentration of 5 × 10^−4^ mol/dm^3^. Measurements were performed for complexes with a molar ratio of 3:1 (ligand/metal). Solutions were obtained by dissolving solid substances in an aqueous solution. The composition of the complexes in aqueous solutions was examined spectrophotometrically (UV) at pH = 7.4.

Spectroscopic and thermal characterisation of the tested complexes was performed in the solid phase. Antioxidant and microbiological tests were performed in aqueous solutions. Before preparing appropriate solutions for testing, analyses of the composition of the complexes in aqueous solutions were performed. The results of these analyses were used to prepare complexes for testing in solutions with an appropriate composition (molar ratio of metal to ligand).

### 2.4. Antioxidant Study

The antioxidant properties of the tested compounds were determined using DPPH (reaction with the 2,2-Diphenyl-1-picrylhydrazylradical), ABTS (reaction with the 2,2’-azino-bis(3-ethylbenzothiazoline-6-sulfonic acid radical) and CUPRAC tests (based on the reduction of Cu^2+^ to Cu^1+^). The assays were performed in accordance with the methodology described in the literature [23,24]. Absorbance measurements were performed using a UV-VIS Carry 5000 spectrophotometer Agilent (Santa Clara, CA, USA). Solutions of Ce(III), Pr(III), Nd(III) and Sm(III) complexes with p-CAH_2_ with a metal–ligand composition of 1:1 were prepared in accordance with the results of previous studies on the composition of the complexes in aqueous solution. First, 10% methanolic solutions of p-CAH_2_ (0.05 and 0.005 mol/L) were added to aqueous sodium hydroxide solution (0.1 mol/L) in a molar ratio of 1:1. Then, aqueous solutions of Ce(III), Pr(III), Nd(III) and Sm(III) chlorides (0.01 mol/L) were prepared and mixed with sodium salts of the ligand (1:1 molar ratio). The final concentration of metal complexes for the determination of DPPH was in the range of 2.00–20.00 mmol/L, while for the ABTS test it was 1.0–16.0 µmol/L and for the CUPRAC test it was 31.25 µmol/L. All experiments were performed with five replicates in three independent experiments and values are expressed as means ± SD.

The antioxidant activity towards the hydroxyl radical was determined spectrophotometrically. The radical was produced by the reaction of Fe^2+^ ions (FeSO_4_) with H_2_O_2_. The radical was then quenched by reaction with salicylic acid, resulting in the coloured compound 2,3-dihydroxybenzoic acid (2,3DHBA) with an absorbance maximum at λ = 510 nm. In the reaction with antioxidant compounds, the amount of the resulting coloured 2,3DHBA acid decreased, which was the basis for the spectrophotometric determination of the quenching capacity of the OH radical. The antioxidant activity was determined for a series of solutions of the tested complexes at a concentration of 0.3–1 mmol/dm^3^.

### 2.5. Minimal Inhibitory Concentration Test

Microorganism commonly found in the environment were selected for this study. They are typically used in basic tests aimed at examining the antimicrobial properties of chemical compounds. A series of dilutions of the tested compounds in sterile Mueller–Hinton agar (Oxoid) were made in order to determine the MIC values. In the next step, the appropriate inoculum of microorganisms was added to each agar medium and incubated at 37 °C for 24 h. *C. albicans* (PCM 2566-FY), *E. coli* (PCM 2857) and *B. subtilis* (PCM 2850) came from the Polish Collection of Microorganisms (Wroclaw, Poland). The microorganisms were grown overnight and then were resuspended in physiological saline to obtain an optical density at 600 nm (OD600) of 0.60, corresponding to 5.0 × 10^8^ CFU/ml. The tested compounds were dissolved in DMSO solution (Sigma-Aldrich). The agar plates with DMSO were the negative controls, while the plates with gentamicin (Sigma-Aldrich, 345815) were the positive control for bacteria, and fluconazole agar plates (Sigma-Aldrich, F-031) were a positive control for fungi. The lowest concentration without visible bacterial growth was determined as the MIC (minimal inhibitory concentration).

### 2.6. Cell Viability of Tested Microorganisms (MTT Test)

*Staphylococcus aureus* (ATCC 25923), *Escherichia coli* (ATCC 25922), *Pseudomonas aeruginosa* (ATCC 27853) and *Candida albicans* (ATCC 10231) were obtained from the ATCC (American Type Culture Collection (Manassas, VA, USA)). The studied strains of microorganisms were cultured in MH II (Mueller–Hinton II broth) for 18 h at 37 °C *(P. aeruginosa, E. coli* and *S. aureus)* and at 26 °C for *C. albicans.* Then, cultures were diluted in fresh MH II to obtain 10^8^ CFU/mL (colony forming units per mL). The inoculum with antimicrobial activities of 10^6^ CFU/mL for the suspension of *P. aeruginosa, E. coli* and *S. aureus* cells and 10^4^ CFU/mL for *C. albicans* cells was used.

The preparation of serial two-fold dilutions of the studied p-CAH_2_ and Ce(III), Pr(III), Nd(III) and Sm(III) complexes was carried out according to Jabłońska-Trypuć et al. [25]. The final concentrations of tested compounds in each well were 2.5 mM, 1.25 mM, 0.63 mM, 0.31 mM, 0.16 mM, 0.08 mM, 0.04 mM, 0.02 mM and 0.01 mM. The study was carried out in aqueous solutions of complexes with a molar ratio of ligand to metal of 1:1.

The antimicrobial activity of p-CAH_2_ and complexes against *C. albicans*, *P. aeruginosa, E. coli* and *S. aureus* was determined using an MTT assay as described in [26]. The antimicrobial activity of the tested compound was expressed as the relative cell viability of *C. albicans*, *P. aeruginosa, E. coli* and *S. aureus* in comparison to the control and presented as a percentage. The determination of antimicrobial activity in all samples was performed in triplicate.

### 2.7. Statistical Analysis

The differences between the obtained means for the relative cell viability of *C. albicans*, *P. aeruginosa, E. coli* and *S. aureus* were evaluated by a one-way analysis of variance (ANOVA) and significant differences were estimated by Dunnett’s test at *p* < 0.05, *p* < 0.01 and *p* < 0.001 using STATISTICA.

## 3. Results and Discussion

### 3.1. Elemental and Thermal Analysis

Table 1 shows the results of the elemental analysis of p-CAH_2_ complexes with Ce(III), Pr(III), Nd (III) and Sm(III). The results showed that, in the solid state, these metals form complexes with p-CAH_2_ in a molar ratio of 1:3 (metal/ligand). The complexes (Ce(III), Pr(III) and Sm(III) cerium, praseodymium and samarium) were hydrated and contained six water molecules each, while the Nd(III) complex contained five water molecules.

**Figure 1:**
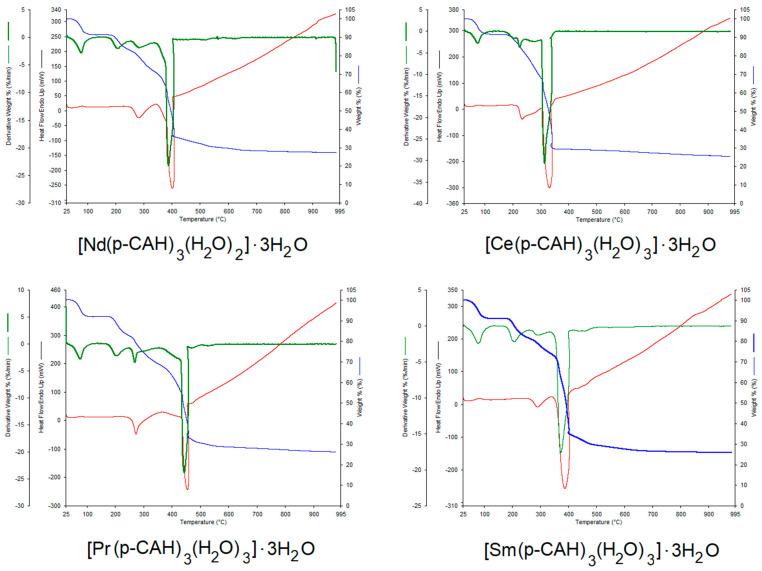
TG, DTG and DSC curves for lanthanide complexes: [Ce(p-CAH)_3_(H_2_O)_3_]·3H_2_O, [Nd(p-CAH)_3_(H_2_O)_2_]·3H_2_O, [Pr(p-CAH)_3_(H_2_O)_3_]·3H_2_O and [Sm(p-CAH)_3_(H_2_O)_3_]·3H_2_O.

The degree of hydration of the obtained complexes was confirmed via thermal analysis of the complexes studied. The thermal analysis made it possible to characterise the type of water present in the molecules. Each of the complexes contained three hydration water molecules and three coordination water molecules. Figure 1 shows the thermal decomposition curves (TG, DTG and DSC) of the p-CAH_2_ complexes with Ce(III), Pr(III), Nd(III) and Sm(III). Table 2 shows the results of the thermal analysis of the studied complexes, i.e., the temperatures of the individual decomposition steps (dehydration, thermal decomposition) and the decomposition products. The first stage of the thermal decomposition of the studied complexes is dehydration. This process occurs at temperatures between 60 °C and 130 °C. In this stage, the complexes lose three hydration water molecules each. The next step in the thermal decomposition of the complexes is the loss of coordination water. This process occurs in the temperature range of 180 to 240 °C. After the loss of water, further heating of the complexes leads to their degradation. All the complexes studied are characterised by a similar thermal stability.

The degradation process of the lanthanide p-coumarates starts immediately after the dehydration process, i.e., at a temperature of about 240 °C. The least thermally stable complex is samarium p-coumarate. Decomposition of this compound starts at 220 °C. The complexes studied undergo thermal decomposition to metal oxides (Ce_2_O_3_, Pr_2_O_3_, Nd_2_O_3_, Sm_2_O_3_). The thermal degradation of lanthanide complexes occurs in several stages, with the individual steps not clearly observed in the degradation curves. One of the first steps in the thermal degradation is probably the decarboxylation of the compounds under investigation, with decomposition of the aromatic ring occurring in a further step. The final products are obtained at temperatures of about 360–400 °C. These products are lanthanide oxides, containing small residual amounts of organic carbon, which undergo slow afterburning to a temperature of about 800 °C (increase in the DSC curve). This is followed by stabilisation of the process—pure lanthanide oxides are formed. The dehydration process of the complexes is endothermic. However, the peaks related to the process of hydration water detachment and coordination were practically invisible in the DSC curves. High-intensity peaks related to the exothermic process of thermal decomposition of the decomposed complexes were observed in the DSC curves. This is related to the combustion of organic carbon in the process of thermal decomposition in an oxygen atmosphere.

### 3.2. IR and Raman Spectra

**Figure 2:**
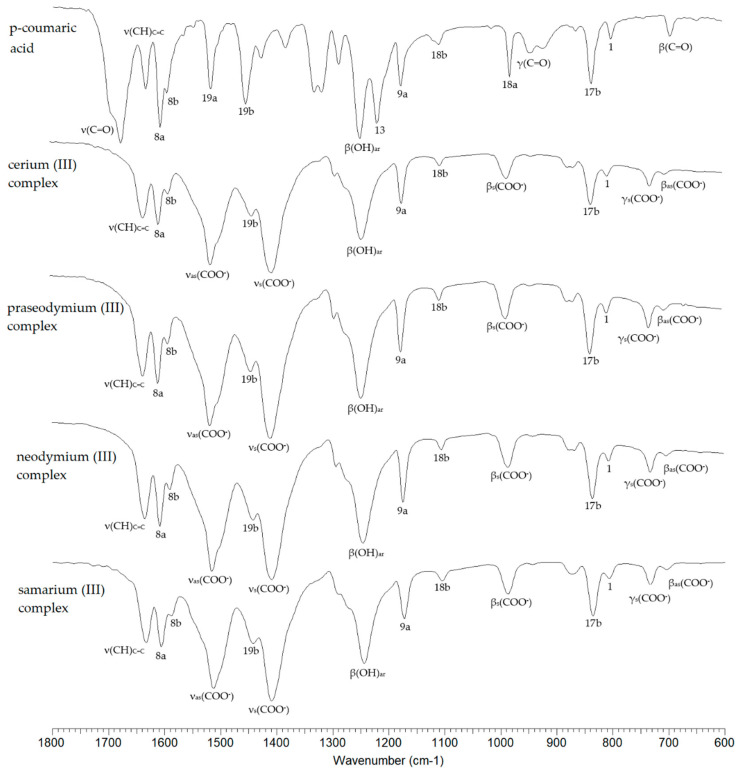
FTIR_KBr_ spectra of p-CAH_2_ and its complexes with Ce(III), Pr(III), Nd(III) and Sm(III).

Figure 2 shows the spectra recorded in the infrared (FTIR KBr) for p-CAH_2_ and its complexes with Ce(III), Pr(III), Nd(III) and Sm(III). The spectra cover the spectral range 600–1800 cm^−^^1^. Selected bands from the vibrations of the carboxyl group/carboxylate anion and the vibration bands of the aromatic system are marked. Table 3 summarises the wavenumbers of the bands present in the FTIR spectra of the studied compounds recorded in a KBr matrix via the ATR and Raman techniques, together with the assignments according to the numbering of normal vibrations given by Versanyi [27]. After complexation of p-CAH_2_, vibrational bands originating from the carboxylate anion are observed in the spectra of the complexes. These are asymmetric stretching vibrations denoted as ν_as_COO^−^ present at 1514–1513 cm^−^^1^ (IR KBr), 1510 cm^−^^1^ (ATR) and 1519–1517 cm^−^^1^ (Raman) and symmetric ν_s_COO^−^ present in the range of 1409–1405 cm^−^^1^ (IR KBr), 1399–1397 cm^−^^1^ (ATR) and 1400–1396 cm^−^^1^ (Raman).

**Figure 3:**
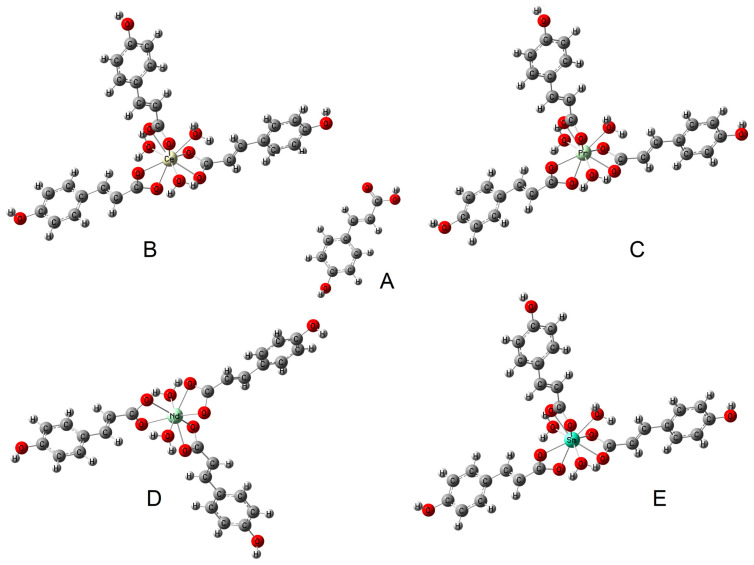
The type of coordination occurring in the studied complexes: (**B**) cerium complex, (**C**) praseodymium complex, (**D**) neodymium complex, (**E**) samarium complex (based on FTIR study) and (**A**) structure of ligand (p-coumaric acid).

The difference in the values of the wavenumbers of the asymmetric and symmetric stretching vibrations of the carboxylate anion (Δν) can indicate the type of metal–ligand coordination in the complex according to the criteria described by Nakamoto [28]. In the complexes studied, this difference is 104–109 cm^−^^1^, which is much lower than the value (Δν) for the sodium salt [29]. This indicates a type of bidentate chelating coordination (Figure 3).

Also present in the spectra of lanthanide complexes of p-CAH_2_ are bands associated with symmetric and asymmetric bending vibrations in the ring plane (β_s_COO^−^ and β_as_COO^−^ vibrations) and bending vibrations outside the ring plane (γCOO^−^). Metal complexation of aromatic carboxylic acids affects the π-electron arrangement of the aromatic ring of the ligand, which consequently affects the reactivity of the chemical compound and its biological activity. Studies have shown that alkali metals destabilise the electron arrangement of the aromatic ring [30], while 3d transition metals and 4f metals can stabilise the electron arrangement of the ligand. Electron system stabilisation is related to electron transfer to the electron system of the metal. This effect can be observed by comparing the infrared and Raman spectra of the ligand and the complex. In 3d transition metal and lanthanide complexes, a shift in the bands towards higher wavenumbers and an increase in band intensities are observed.

In the case of the spectra of Ce(III), Pr(III), Nd(III) and Sm(III) complexes, an increase in the wavenumbers of most of the bands of the aromatic system relative to the ligand was observed. Here, we mention the bands numbered 20b, 8a, 17a, 10a, 17b, 1. The bands numbered 9a and 18b do not change position in the spectra of the complexes relative to the spectrum of the ligand. A few bands in the spectra of the complexes are shifted towards higher wavenumber values relative to their position in the spectrum of p-CAH_2_ (7b, 8b, 19b). The change in the position of the bands confirms that in the case of complexation of p-CAH_2_ with lanthanide ions (Ce(III), Pr(III), Nd(III) and Sm(III)), an increase in the stability of the electron arrangement of the aromatic ring of the ligand is observed. An increase in the aromaticity of the ring reduces its reactivity in substitution reactions. At the same time, a change in the electron charge distribution of the aromatic ring will affect the electron density distribution of the hydroxyl group directly attached to the aromatic system, which will affect the antioxidant activity of lanthanide complexes with p-CAH_2_.

**Table 3:** Wavenumbers of the bands present in the FTIR spectra of p-CAH_2_ and its complexes with Ce(III), Pr(III), Nd(III) and Sm(III) recorded via KBr matrix, ATR and Raman techniques.

p-CAH_2_ [29]			[Ce(p-CAH)_3_(H_2_O)_3_]·3H_2_O			[Pr(p-CAH)_3_(H_2_O)_3_]·3H_2_O			[Nd(p-CAH)_3_(H_2_O)_2_]·3H_2_O			[Pr(p-CAH)_3_(H_2_O)_3_]·3H_2_O			Assignment	No. [27]
IR_KBr_	IR_ATR_	Raman	IR_KBr_	IR_ATR_	Raman	IR_KBr_	IR_ATR_	Raman	IR_KBr_	IR_ATR_	Raman	IR_KBr_	IR_ATR_	Raman		
3383 s	3375 w	-	3394 m	-	-	3422 m	-	-	3378 m	-	-	3357 m	-	-	ν(OH)_ar_	-
-	-	-	3256 m	-	-	3251 m	-	-	3251 m	-	-	3256 m	-	-	ν(OH)_ar_	-
3026 w	-	3025 vw	3024 w	-	-	3027 w	-	3024 vw	3025 w	-	-	3027 w	-	-	ν(CH) + ν(CH)_C=C_	20b
2959 m	-	-	2955 w	-	-	2952 vw	-	-	3952 vw	-	-	2955 vw	-	-	ν(CH + ν(CH)_C=C_	7b
2839–2513 m	-	-													ν(OH)	-
1672 vs	1667 vs	-	-	-	-	-	-	-	-	-	-	-	-	-	ν(C=O)	-
1628 s	1626 m	1636 m	1633 s	1633 m	1635 s	1633 s	1633 m	1636 s	1633 s	1633 s	1636 s	1633 s	1633 m	1633 s	ν(CC)_C=C_	-
1601 vs	1600 s	1606 vs	1606 s	1603 m	1607 vs	1606 s	1603 m	1608 vs	1606 s	1605 s	1607 vs	1606 s	1605 m	1605 vs	ν(CC)	8a
1591 s	1589 s	1593 m	1589 m	-	-	1589 m	-	-	1589 m	-	-	1588 m	-	-	ν(CC)	8b
1512 s	1510 m	1519 w	-	-	1547 w	-	-	-	-	-	1547 w	-	-	-	ν(CC)	19a
-	-	-	1514 vs	1510 s	1517 w	1514 vs	1510 vs	1517 w	1514 vs	1510 vs	1519 w	1513 vs	1510 vs	1518 w	ν_as_(COO)	-
1449 vs	1446 s	1448 w	1440 m	1440 m	1435 w	1440 m	1443 m	1433 w	1440 s	1441 s	1435 w	1442 m	1439 m	1439 w	ν(CC)	19b
1422 s	1422 m	-	-	-	-	-	-	-	-	-	-	-	-	-	β(CH)_C=C_	-
-	-	-	1405 vs	1397 vs	1397 w	1405 vs	1398 vs	1400 w	1406 vs	1399 vs	1396 w	1409 vs	1399 vs	1400 w	ν_s_(COO-)	-
1379 m	1376 m	-													β(CH)_C=C_ + β(OH)_ar_	14
1314 s		1306 w	1322 vw	-	1321 vw	-	-	1320 w	-	-	1321 vw	-	-	1323 vw	β(CH)_C=C_ + β(OH)_ar_	3
1283 m	1309 s	1282 w	1292 m	-	1292 vw	1292 w	-	1293 vw	1292 m	-	1291 vw	1289 sh	-	1293 vw	ν(C-OH)	
1244 vs	1241 vs	1260 m	1244 s	1239 s	1249 m	1244 s	1239 s	1249 m	1244 s	1241 vs	1250 m	1244 s	1239 s	1251 m	β(OH)	-
1215 s	1211 s	1212 w	-	-	1204 w	-	-	1204 w	-	-	1202 w	-	-	1205 w	β(CH)	13
1173 s	1171 s	1171 w	1173 m	1171 m	1171 m	1173 m	1171 m	1171 w	1173 m	1171 s	1171 w	1173 m	1171 m	1170 m	β(CH)	9a
1105 m	1103 m	-	1105 w	1106 w	-	1105 w	1109 w	-	1105 w	1106 s	-	1105 w	1106 m	-	β(CH)	18b
1013 w	1012 w	-													β(CH)	18a
			986 m	986 w	984 vw	986 w	986 m	985 vw	984 m	986 s	987 vw	987 w	986 m	980 w	β_s_(COO)	
978 s	977 s	977 vw													ν(CCO)	
941 m	937 m	952 vw	943 w	-	-	-	-	-	942 w	942 s	-	943 vw	-	-	γ(CH)	17a
920 s	912 m	-	-	-	-	-	-	-							γ(CO)	-
-	-	-	877 m	873 w	-	877 w	873 w	-	875 w	873 s	-	873 w	870 m	867 w	γ(CH)	5
860 w	-	864 w	867 m	-	867 vw	867 w	-	866 vw	867 w	-	867 vw	-	-	-	γ(CH)	10a
833 s	827 s	837 w	836 s	834 m	837 vw	836 m	834 m	-	834 m	834 s	-	836 m	834 s	-	γ(CH)	17b
799 m	797 m	800 w	806 m	805 w	806 vw	806 w	807 m	806 vw	806 m	-	807 vw	806 w	807 m	806 w	α(CCC)	1
-	-	-	731 m	728 m	-	731 m	728 m	-	731 m	731 s	-	732 w	732 m	732 vw	γ_s_(COO)	-
			704 m	-	-	704 w	704 m	-	703 w	700 s	-	704 vw	703 m	706 vw	β_as_(COO)	-
692 m	690 m		-	-	-	-	-	-	-	-	-	-	-	-	β(CO)	-
646 w	645 w	645 vw	643 w	-	642 vw	-	-	643 vw	642 w	-	643 vw	643 vw	630 m	642 vw	φ(CC)	4

### 3.3. UV Spectra

**Figure 4:**
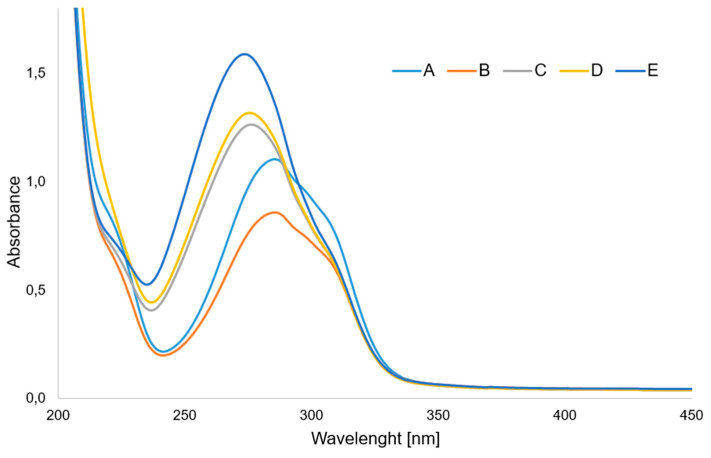
UV spectra of aqueous solution of (**A**) p-CAH_2_ and its complexes with (**B**) Ce(III), (**C**) Sm(III), (**D**) Pr(III) and (**E**) Nd(III). (Molar ratio of ligand to metal—3:1).

Figure 4 shows the UV spectra of aqueous solutions of p-CAH_2_ and its complexes with Ce(III), Pr(III), Nd (III) and Sm(III). In the UV spectrum of p-CAH_2_ recorded in aqueous solution at a concentration of 5 × 10^−5^ mol/dm^3^ in the range 200–450 nm, one band is observed, the absorption maximum of which is located at a wavelength of λ_max_ = 285.5 nm. This band is responsible for the electron transition in the ligand’s π→π* aromatic ring. In the spectra of light lanthanide complexes with p-CAH_2_, this band undergoes a hypsochromic shift. For the cerium complex, this band is located at λ_max_ = 285.0 nm; for samarium, λ_max_ = 276.0 nm; for praseodymium, λ_max_ = 275.5 nm; and for the neodymium complex, λ_max_ = 273.5 nm. Analysis of the UV spectra showed that there is a shift in the absorption maxima of the π→π* band in the spectra of the complexes relative to the band present in the spectrum of the ligand. A change in the intensity of these bands in individual complexes was also observed (generally, an increase in intensity). This proves the influence of the lanthanide ion on the aromatic system of the ligand (changes in the position of the π→π*band), as well as on the change in the distribution of the electron density of the hydroxyl group substituted on the aromatic ring. In turn, this affects the ability of hydroxyl group to react with free radicals. Light lanthanide ions are known to have similar physicochemical properties; therefore, they have a similar effect on the electron system of the ligand. Therefore, the antioxidant activity of the complexes is at a similar level. The UV spectra of solutions of lanthanide complexes with different metal/ligand molar ratios were also recorded. This was important because antioxidant and microbiological tests were performed on these compounds in solutions.

**Figure 5:**
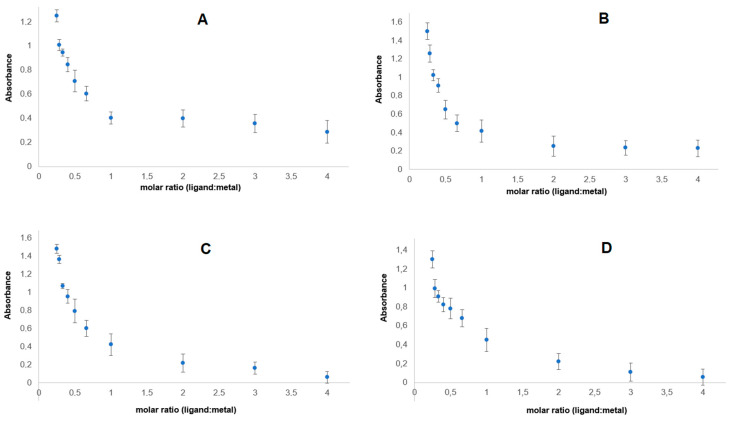
Dependence of absorbance on the composition of lanthanide complexes of p-CAH_2_ with (**A**) Ce(III), (**B**) Nd(III), (**C**) Pr(III) and (**D**) Sm(III).

Figure 5 shows the dependence of the absorbance of lanthanide complexes with p-CAH_2_ on the composition of the complex in aqueous solutions. The course of the curves indicates that at concentrations of 5 × 10^−5^ mol/dm^3^, metal–ligand complexes are formed in a molar ratio of 1:1. This study was carried out in aqueous solutions of tris HCl buffer with pH = 7.4.

### 3.4. Antioxidant Properties

**Figure 6:**
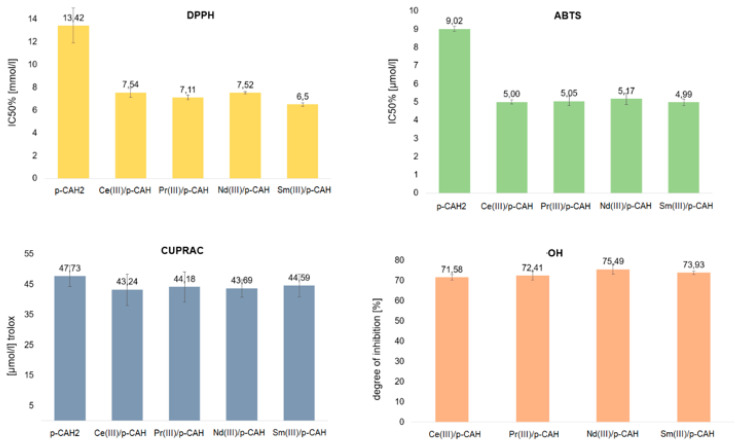
Antioxidant activity of p-CAH_2_ and its complexes with Ce(III), Pr(III), Nd(III) and Sm(III) determined by DPPH, ABTS, CUPRAC and OH assays. (Molar ratio of ligand to metal—1:1).

The antioxidant properties of Ce(III), Nd(III), Pr(III) and Sm(III) complexes of p-CAH_2_ were studied by means of four different spectroscopic assays. The final concentration of metal complexes for the determination of DPPH was in the range of 2.00–20.00 mmol/L, while for the ABTS test it was 1.0–16.0 µmol/L and for the CUPRAC test it was 31.25 µmol/L. The molar ratio of metal to ligand was 1:1, which is consistent with the results obtained in Job’s study. The results of the tests carried out are shown in Figure 6.

All complexes of p-CAH_2_ showed higher antioxidant properties than the free ligand. DPPH and ABTS assays are related to mixed HAT (hydrogen atom transfer) and SET (single-electron transfer) mechanisms that are generally called SPLET (sequential proton loss–electron transfer) or SET-PT (single-electron transfer–proton transfer) mechanisms [31]. In these tests, the tested complexes were characterised by a nearly two times higher antioxidant activity than the free ligand. However, it should be noted that the activity of individual complexes remains at a fairly similar level. The CUPRAC methods rely on the reduction of metal ions Cu^2+^ in the presence of substances with antioxidant properties.

The measurement of the reducing capacity of a given chemical compound was carried out spectrophotometrically for the complex compounds formed with reduced ions. The antioxidant activity in the CUPRAC assay is expressed as Trolox equivalents. In the CUPRAC assay, the Ce(III), Nd(III), Pr(III) and Sm(III) complexes with p-CAH_2_ were shown to have a lower reducing capacity than the free ligand. However, the decrease in the reducing capacity of the complexes with respect to the ligand was small and was within the statistical error limit.

The Cu^2+^ cation reduction method (CUPRAC) and the reaction with the hydroxyl radical are based on different mechanisms of antiradical activity. In the case of the reduction of Cu^2+^ cations, no significant difference was noted between the effects of the ligand and the complexes.

Studies of antioxidant activity against the hydroxyl radical OH have shown that lanthanide complexes with p-CAH_2_ at a concentration of 0.5 mmol inhibit the action of the radical by approximately 70% (Table 4). The highest degree of inhibition is shown by the neodymium complex (75.5%) and the lowest by the cerium complex (71.6%). In the case of p-CAH_2_, no OH radical inhibitory activity was observed in the range of complex concentrations tested. p-CAH_2_ exhibits a significantly lower antioxidant activity against the OH radical than that of the tested complexes. In the range of the tested concentrations of lanthanide complexes with inhibitory effects on OH radicals, p-CAH_2_ did not show such properties.

### 3.5. Antimicrobial Properties

Table 5 shows minimal inhibitory concentration (MIC) of p-CAH_2_ and its complexes with Ce(III), Pr(III), Nd(III) and Sm(III), as well as the minimal inhibitory concentration (MIC) values for antibiotics against selected microorganisms. *C. albicans* shows the greatest resistance to both p-CAH_2_ and its complexes with metals ions in comparison to Gram-positive and Gram-negative bacteria. It has been shown that p-CAH_2_ has a strong limiting effect on the growth of pathogenic fungi and stimulates numerous defence mechanisms in host cells [32]. However, *C. albicans* is known to be a pathogen that forms multimicrobial biofilms with various bacteria. These biofilms are formed by communities of microorganisms belonging to different kingdoms. These microorganisms interact with each other through various molecular mechanisms [33]. For this reason, this yeast very often shows a high resistance to various antifungal substances, which makes it difficult to treat fungal infections. Complexes of p-CAH_2_ with metal ions have a much stronger fungicidal and bactericidal effect. Complexes of metal ions with various organic compounds have been widely considered as potential antifungal and antibacterial drugs for some time. Metal complexes with amino acids have been shown to have an antibacterial effect against both Gram-positive and Gram-negative bacteria and against fungal pathogens [34]. In addition, metal nanoparticles are used as carriers of antifungal drugs to increase their biocompatibility and effectiveness [35]. As a result of the conducted analyses, it was shown that metal ions significantly increase the growth-inhibiting properties of p-CAH_2_ in relation to yeast, as well as Gram-positive and Gram-negative bacteria.

### 3.6. Cell Viability of Tested Microorganisms

**Figure 7:**
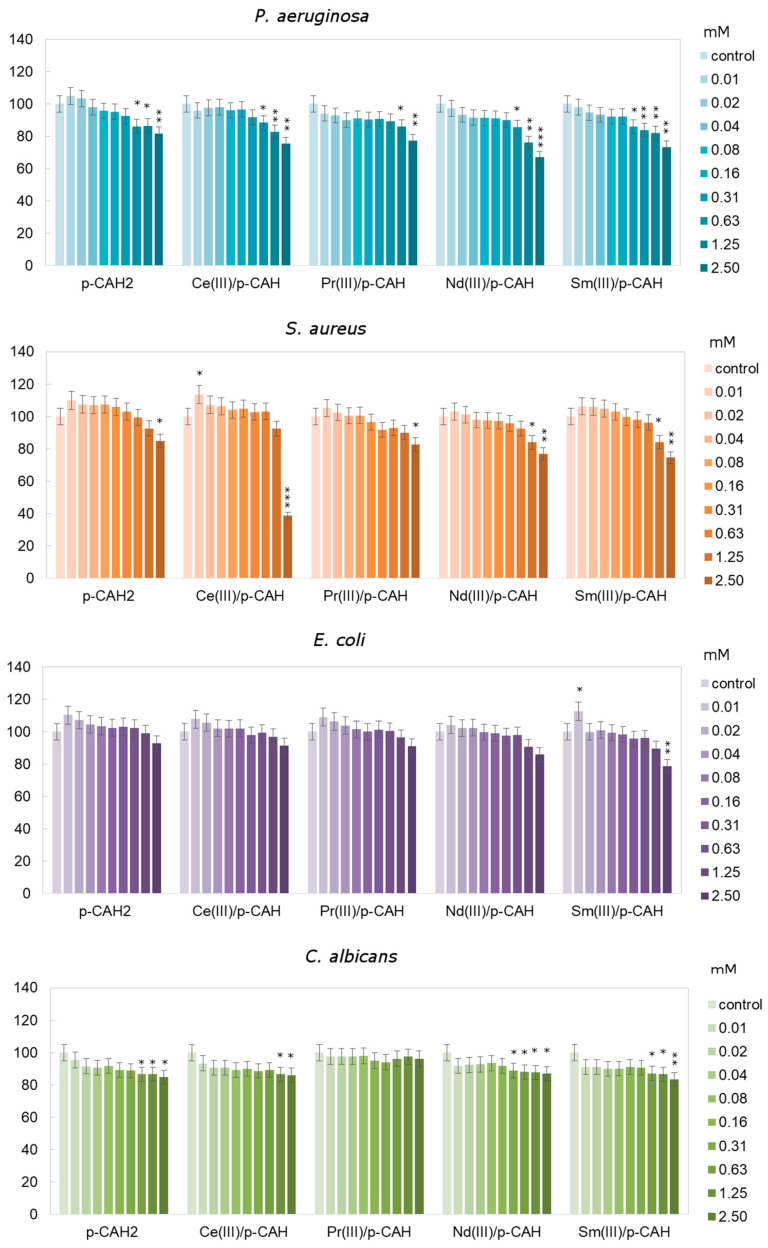
Different relative cell viability of *C. albicans*, *P. aeruginosa*, *E. coli* and *S. aureus* after 24 h depending on the concentration of p-CAH_2_ and its complexes with Ce(III), Pr(III), Nd(III) and Sm(III) (*—*p* < 0.05, **—*p* < 0.01 and ***—*p* < 0.001 indicate significant differences between treatments and control, which were evaluated via Dunnett’s test; error bars represent standard deviation ± SD). (Molar ratio of ligand to metal—1:1).

To determine the effect of p-CAH_2_ and its complexes with Ce(III), Pr(III), Nd(III) and Sm(III) on *E. coli, C. albicans, S. aureus* and *P. aeruginosa*, an MTT assay was used. As presented in Figure 7, in the case of *C. albicans*, it was observed that p-CAH_2_ and its complexes with Ce(III), Pr(III), Nd(III) and Sm(III) caused an insignificant decrease in microorganism growth with an increase in the concentration of the studied acids, where the highest value was approximately 20%. The highest inhibition for *C. albicans* was observed after samarium and neodymium complex application in the concentration range of 0.63 to 2.5 mM (Figure 7).

For p-CAH_2_ and its complexes with Ce(III), Pr(III), Nd(III) and Sm(III), the average relative viability of *P. aeruginosa* was approximately between 1 and 18% compared to untreated cells. After the application of acid complexes at concentrations of 1.25 to 2.5 mM, a significant decrease in cell viability of approximately of 19 to 31% was observed compared to the control cells.

The result obtained for *S. aureus* showed that p-CAH_2_ and its complexes with Ce(III), Pr(III), Nd(III) and Sm(III) caused inhibition of the growth of the tested bacteria with increasing concentrations of the analysed acid. It was observed that the highest level of inhibition (>50%) was obtained for 2.5 mM of the cerium complex compared to the control cells. Moreover, the application of p-CAH_2_ and the cerium complex significantly increased the number of *S. aureus* in the concentration range of 0.01 to 0.31 mM by approximately 7%.

After application of the complexes on *E. coli* at the lowest concentrations (from 0.01 to 0.16 mM), an increase in relative cell viability of 1 to 12% was observed. At concentrations of 0.31 to 2.5 mM, there was a decrease in the relative number of viable cells of 1 to 22% compared to the untreated control cells (Figure 7).

## 4. Conclusions

P-coumaric acid is a compound of natural origin found in many plants (fruits, vegetables, herbs). It is characterised by high antioxidant and antimicrobial potentials. Complexing the acid with metal ions may change its activity. Using lanthanide ions in paracoumaric acid complexes stabilises the aromatic system in the ligand, which was observed by analysing the infrared and Raman spectra of the tested compounds. Changing the electronic charge distribution in the aromatic ring increases the antioxidant activity by activating the hydroxyl group, which participates in radical quenching processes. In solid-phase lanthanide complexes (cerium, samarium, praseodymium and neodymium), the central atom is attached to two molecules of p-coumaric acid through its carboxyl groups. Thermal analysis showed that the complexes were hydrated and had a higher thermal stability than the ligand. Studies have shown that complexation of p-coumaric acid changes its biological activities. Lanthanide complexes (praseodymium, neodymium, samarium and cerium) showed almost twice the antioxidant activity in tests with ABTS and DPPH radicals. In studies of antioxidant activity with the hydroxyl radical, the complexes showed a much stronger radical inhibiting effect than the uncomplexed ligand. Complexing p-coumaric acid with lanthanide ions also affects its antimicrobial properties. Complexes of p-CAH_2_ with metal ions have a much stronger fungicidal and bactericidal effect. Lanthanide complexes with biologically derived ligands are of increasing interest due to their many beneficial properties. There are many reports in the literature showing that these compounds have high antimicrobial, antioxidant and even anticancer potentials. The results of the analyses carried out in this work and in our other works have shown that complexes of lanthanides with phenolic acids are compounds that could be potentially used as biologically active agents. Complexing lanthanide ions with aromatic acids stabilises the aromatic system, thereby influencing the reactivity and biological activity of these compounds.

## Figures and Tables

**Table 1:** Results of elemental analysis of p-CAH_2_ complexes with Ce(III), Pr(III), Nd(III) and Sm(III).

Formula	Experimental		Theoretical	
	%C	%H	%C	%H
[Ce(CAH)_3_(H_2_O)_3_]·3H_2_O	43.45	4.42	43.96	4.51
[Pr(p-CAH)_3_(H_2_O)_3_]·3H_2_O	43.74	4.34	43.91	4.50
Nd(p-CAH)_3_(H_2_O)_2_]·3H_2_O	43.62	434	43.72	4.48
[Sm(p-CAH)_3_(H_2_O)_3_]·3H_2_O	43.97	400	43.36	4.45

**Table 2:** Thermal decomposition parameters (TG, DTG and DSC) of p-CAH_2_ and its complexes with Ce(III), Pr(III), Nd(III) and Sm(III).

Compound	Stage	TG T_range_ [°C]	DTG (DSC) Tmax. Peaks [°C]	Peak Nature	Mass Loss [%]		Final Residue
					Calc.	Found	
p-CAH_2_	decomposition	180–550	225.5 (221.8)	endo	100%	2.3%	-
[Ce(p-CAH)_3_(H_2_O)_3_]·3H_2_O	dehydration	75–120	74.5	endo	7.89%	8.29%	[Ce(p-CAH)_3_(H_2_O)_3_]
	dehydration	180–240	224.89	endo	14.64%	15.23%	Ce(p-CAH)_3_
	decomposition	260–380	314.64 (331.26)	egzo	73.69%	74.51%	Ce_2_O_3_
[Pr(p-CAH)_3_(H_2_O)_3_]·3H_2_O	dehydration	75–130	76.99	endo	7.89%	7.86%	[Pr(p-CAH)_3_(H_2_O)_3_]
	dehydration	180–260	212.35	endo	14.62%	15.11%	Pr(p-CAH)_3_
	decomposition	260–460	443.69 (454.26)	egzo	73.59%	73.75%	Pr_2_O_3_
Nd(p-CAH)_3_(H_2_O)_2_]·3H_2_O	dehydration	60–120	78.09	endo	7.85%	8.56%	[Nd(p-CAH)_3_·(H_2_O)_2_]
	dehydration	180–240	207.38	endo	14.56%	15.45%	Nd(p-CAH)_3_
	decomposition	260–410	388.26 (401.94)	egzo	73.15%	72.72%	Nd_2_O_3_
[Sm(p-CAH)_3_(H_2_O)_3_]·3H_2_O	dehydration	60–120	76.99	endo	7.78%	7.86%	[Sm(p-CAH)_3_(H_2_O)]
	dehydration	175–220	208.10	endo	14.44%	15.46%	Sm(p-CAH)_3_
	decomposition	240–460	443.69 (454.26)	egzo	72.68%	74.75%	Sm_2_O_3_

**Table 4:** Antioxidant activity of p-CAH_2_ and its complexes with Ce(III), Pr(III), Nd(III) and Sm(III) determined by DPPH, ABTS, CUPRAC and OH assays. (Molar ratio of ligand to metal—1:1).

	DPPH	SD *	ABTS	SD	CUPRAC	SD	·OH	SD
p-CAH	13.42	1.54	9.02	0.13	47.73	3.44	-	-
Ce(III)/pCAH	7.54	0.4	5.00	0.12	43.24	5.20	71.58	1.33
Pr(III)/pCAH	7.11	0.2	5.05	0.26	44.18	4.94	72.41	2.15
Nd(III)/pCAH	7.52	0.1	5.17	0.30	43.69	2.88	75.49	2.38
Sm(III)/pCAH	60.5	0.16	4.99	0.19	44.59	3.76	73.93	0.87

* SD—standard deviation.

**Table 5:** Minimal inhibitory concentration (MIC) of p-CAH_2_ and its complexes with Ce(III), Pr(III), Nd(III) and Sm(III) for selected strains of microorganisms [mmol/L].

Species of Microorganism	Compound						
	p-CAH_2_	Ce(III)/p-CAH	Pr(III)/CAH	Nd(III)/p-CAH	Sm(III)/p-CAH	Fluconazole	Gentamicin
	MIC (mmol/L)						
*Escherichia coli*	42.91	6.6	6.58	6.51	6.39	-	1.05
*Candida albicans*	49.03	13.20	13.15	13.03	15.97	0.82	-
*Bacillus subtilis*	42.91	6.6	9.86	9.77	6.39	-	10.50

## Data Availability

The data presented in this study are available upon request from the corresponding author.
